# Housing displacement experiences following major flooding in regional Australia: A qualitative study of online news articles

**DOI:** 10.1371/journal.pone.0357805

**Published:** 2026-09-09

**Authors:** Jessica Chen, Rebecca McNaught, Jodie Bailie, Kanchana Ekanayake, Ross Bailie

**Affiliations:** 1 School of Public Health, The University of Sydney, Sydney, New South Wales, Australia; 2 School of Health Sciences, The University of Sydney, Sydney, New South Wales, Australia; 3 University Centre for Rural Health, The University of Sydney, Lismore, New South Wales, Australia; 4 Centre for Disability Research and Policy, The University of Sydney, Sydney, New South Wales, Australia; 5 University of Sydney Library, The University of Sydney, Sydney, New South Wales, Australia; Shanghai University, CHINA

## Abstract

**Background:**

The health impacts of flood displacement are increasingly acknowledged within broader literature. However, there remains limited research on the challenges of accessing post-flood accommodation and how these influence health and wellbeing, particularly mental health. This study explores the nature and extent of media reporting on 1) the experiences of people displaced from their homes and 2) the influence of accommodation access on physical and mental health and wellbeing, following a major flood in regional Australia. We then reflect on how accurately media reporting reflects broader research available regarding the impacts of flood displacement upon various population groups.

**Methods:**

We conducted a novel qualitative study of online news articles on displacement following flooding in Lismore, Australia in 2022. Relevant articles published over two years were identified through database searches. Data were inductively analysed using content analysis.

**Results:**

The search yielded seventy-one online news articles which met the inclusion criteria. Poor mental health outcomes were associated with displacement, stemming from reports of traumatisation, distress and feeling “in limbo.” Online news articles reported on experiences of various emergency, short-term and long-term housing measures, and insights into the factors which influence housing related decision-making across these time periods in flood-affected communities. This includes utilisation of formal government programs, and informal housing arrangements which may not be captured in formal reporting. There was a relative lack of attention to the experiences of the most severely affected groups and their needs.

**Conclusion:**

We found that news reporting highlights the deleterious influence of flood displacement on health, which supports existing literature. However, there appears to be limited focus on challenges faced by priority populations within news reporting. Results indicate that poor mental health outcomes in displaced people may be improved through enhanced communication and collaboration between communities and governments during flood recovery and disaster displacement planning.

## Introduction

Climate change is affecting the frequency and magnitude of flood events globally [1,2]. Flooding causes significant harm to individuals and communities, particularly those who become displaced, both temporarily and long-term [3,4]. Displacement refers to “situations where people are forced… to leave their homes or places of habitual residence as a result of a disaster or to avoid the impact of an immediate and foreseeable natural hazard” [5p12].

Previous research has identified numerous physical and mental health impacts linked with disaster-related displacement [6,7]. For example, displaced populations experience increased risk of infectious and non-communicable diseases, difficulties accessing healthcare, poor social determinants of health, and poor psychosocial or mental health outcomes [6–9].

Socially vulnerable populations are disproportionately impacted by disasters such as flooding, including increased likelihood of displacement [10,11]. Viewed from a social vulnerability perspective, these unequal disaster-related consequences are experienced by different populations because of pre-existing disparities, such as socioeconomic status [12,13].

Decision-making processes regarding post-flood housing, including in relation to remaining in or relocating from flood-affected areas, and the impact of these choices on wellbeing have been studied [14,15]. However, there has been limited exploration of the experience involved in securing other housing and accommodation options, such as temporary accommodation and informal housing arrangements. There has also been limited exploration into the experiences of flood-related displacement for various population groups such as elderly people, people with disabilities, single-parent families, children, and Indigenous peoples despite research indicating these populations may be disproportionately affected by disasters [16].

News articles reflect issues that attract media attention over various time periods [17] and therefore may provide useful insights regarding experiences of displacement and disaster responses. However, we found most research on media reporting of disasters has focused on tone and style of reporting, rather than exploring the usefulness of news articles as a source of information [18–20]. Analysis of news articles can be a relatively cost-effective method of gathering information regarding experiences and narratives in instances where more traditional qualitative research methods, such as interviewing, may not be appropriate or feasible [17,21].

Regional and remote areas face numerous challenges with disaster recovery due to limited financial resources and high social vulnerability, which can lead to widening socioeconomic and health inequities [22,23]. Given the likelihood of increased flooding events, it is important to improve understanding of the experiences of those displaced in regional and remote areas to inform future recovery efforts and to guide further research [23]. It is also important to ensure reporting of flood impacts reflects the relative impacts for different groups.

The aim of this study is twofold. Firstly, we aim to examine news reporting of the experiences of those displaced from their homes as a result of flooding in a regional area and the health and wellbeing implications associated with accessing and securing emergency, temporary and longer-term accommodation and housing. Secondly, we aim to determine whether news reporting accurately reflects findings from other research regarding the experiences and impacts of displacement on various population groups. In doing so, we hope to better understand the linkages between displacement, and health and wellbeing within post-flood communities, and the usefulness of media reporting as an information source in disaster research.

### Study setting

**Fig 1:**
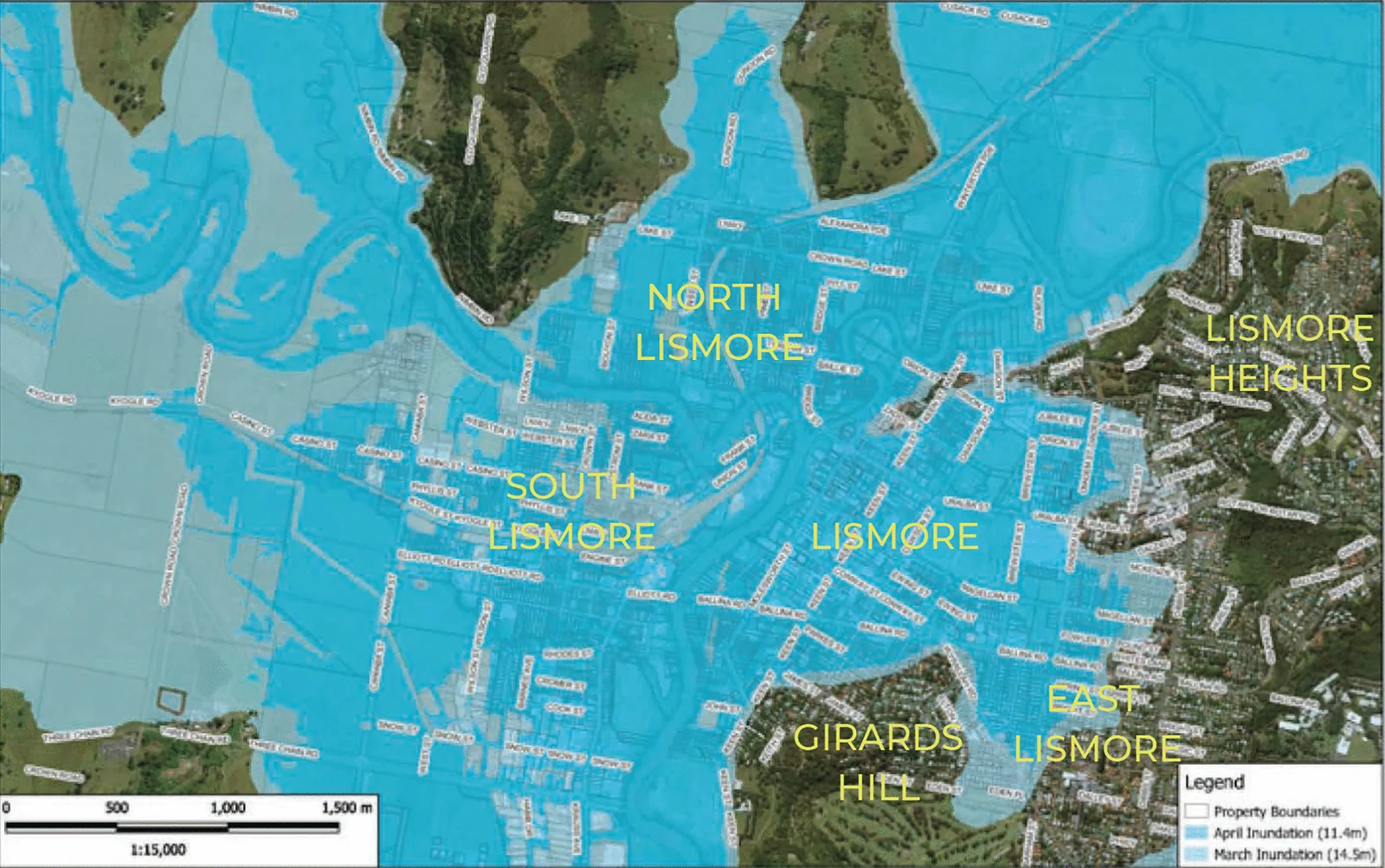
Map of Lismore with 2022 flooding levels indicated. Reprinted from Lismore City Council [27] under a CC BY license, with permission from Lismore City Council, original copyright 2022.

Lismore is an inner regional Australian local government areas (LGA) located in the Northern Rivers region, a highly flood-prone area of New South Wales (NSW) [24,25]. In 2022, Lismore was affected by two floods on 28 February and 29 March, with damage repairs for the Lismore LGA alone estimated at approximately $1 billion [24,26]. Significant parts of Lismore were affected by the floods, however South Lismore and North Lismore were found to be the most flood-impacted suburbs [27] (see Fig 1). On 28 February 2022, flood waters rose to 14.4m-high, over 2m higher than any prior floods recorded in Lismore, resulting in over-topping of levees which had, in prior floods, protected the Lismore CBD and South Lismore areas from significant flood damage [24]. The overall impact of the 2022 floods on the Lismore LGA was substantial, with reports that 1,720 residential properties sustained flood damage and an estimated 2,000 people were displaced [27].

Lismore is considered to have high social vulnerability, and higher numbers of single-parent families, renters, Aboriginal and Torres Strait Islander peoples, low-income earners and lower educational attainment compared to the general population of NSW [28]. The 2017 Lismore flood demonstrates previous inequities in impacts across various population groups [11,12,29]. For example, residents from socio-economically marginalised population groups were more likely to have experienced flooding and subsequent displacement [29] with 82% of flood-affected residents in the Lismore Town Centre belonging to the lowest socio-economic quintile [12]. Furthermore, people with disability were more likely than others to be evacuated, experience lengthy displacement and have poorer mental health outcomes [11]. Lismore provides a case study of the experience of displacement in regional and rural areas which may be of relevance to other similarly situated flood-prone areas with high social vulnerability.

## Methods

### Study design

We conducted a qualitative study of online news articles [17]. We adopted a constructivist perspective, which assumes that neither data nor theories are discovered but rather are constructed based on shared experiences [30]. Reporting of our study was guided by the ‘Consolidated criteria for Reporting Qualitative research’ (COREQ) guidelines [31]. Three of the four authors are researchers based in Lismore and personally experienced the flooding events of 2022, giving them unique insights into context [32]. The first author was based in Sydney, however, the lived experience of the other authors guided the direction of our research and the level of importance placed on some topics during study design and analysis. In particular, analysis of reporting on priority populations and the focus on extracting information regarding informal and formal accommodation arrangements was guided by the authors’ lived experience and former disaster research in the region. For further information about authors, see note following the conclusion of this paper. Ethics approval was not required as we used publicly available data. Identifying information of people mentioned in news articles was redacted or removed wherever necessary to preserve privacy and confidentiality.

### Search strategy

News articles were identified by searching Google News, which was selected after consultation with an academic librarian (KE). Trial searches of other databases returned limited relevant articles. A timeframe of 28 February 2022–28 February 2024 was selected to allow analysis of online news reporting over the 2-year period post-flood. Online news articles refer to written articles published by news outlets on their websites or websites of their associated parent company. The search was conducted on 4 April 2024 using the key search terms ‘flood’, ‘2022’, ‘home OR house’ and ‘Lismore’.

Results were then screened against inclusion criteria and excluded from analysis if they: 1) were not an online news article; 2) contained no reference to the 2022 NSW Floods; 3) did not discuss Lismore; 4) did not discuss displacement and/or post-flood housing and accommodation; and 5) were not accessible for free through the University of Sydney library or unpaid subscriptions.

### Data extraction

A data extraction tool was developed in Microsoft Excel, covering variables such as date of publication, headline title, author, and publisher. Other categories included: topics discussed, population groups mentioned, accommodation types and solutions discussed, factors associated with decision-making, government and community organisations discussed, experiences and opinions in accessing accommodation types and assistance, and influences on mental health concepts. The extraction tool was piloted on 10 articles and refined before applying to the rest. One reviewer (JC) extracted data from the articles and results were checked by one other reviewer (RM).

### Data analysis

Data were inductively analysed in a recursive process that followed the steps of content analysis outlined by Elo and Kyngas [33]: 1) JC read and reread the data to get a sense of the whole dataset, and to gain a deeper understanding of the experiences of displacement reported, while making reflection notes during the process; 2) JC open coded the data writing notes and headings that described the content; 3) JC developed a set of categories by combining concepts with similar meanings; 4) JC conferred with RM, RB and JB on the initial set of categories derived, resolving disagreements through discussion. Throughout this process JC conferred with the authorship team to discuss categorisation. To ensure consistency, all authors, drawing on their experience, checked the results against their understanding of displacement. The occurrence of specific terms was counted to identify patterns in the data, helping to determine the prominence of different themes.

## Results

The search yielded 98 articles. After duplicate removal and screening 71 online news articles (S1 File) met the inclusion criteria from across 12 news outlets (Table 1). Approximately two-thirds of all online news articles were published across two news outlets, ABC News (n = 25) and The Guardian (n = 22) (Table 1).

**Table 1:** Online news articles published by news outlet, between 28 February 2022 and 28 February 2024, *n = number of individual articles.*

Online news outlet	No. of articles (n = 71)	%
ABC News	25	35
The Guardian	22	31
The Lismore Northern Star (accessed via The Daily Telegraph)	6	8
9 News	4	6
Sydney Morning Herald	3	4
The Conversation	2	3
SBS News	2	3
Echonetdaily	2	3
World Socialist Website	2	3
BroadAgenda	1	1
The Monthly	1	1
The Daily Telegraph	1	1

**Fig 2:**
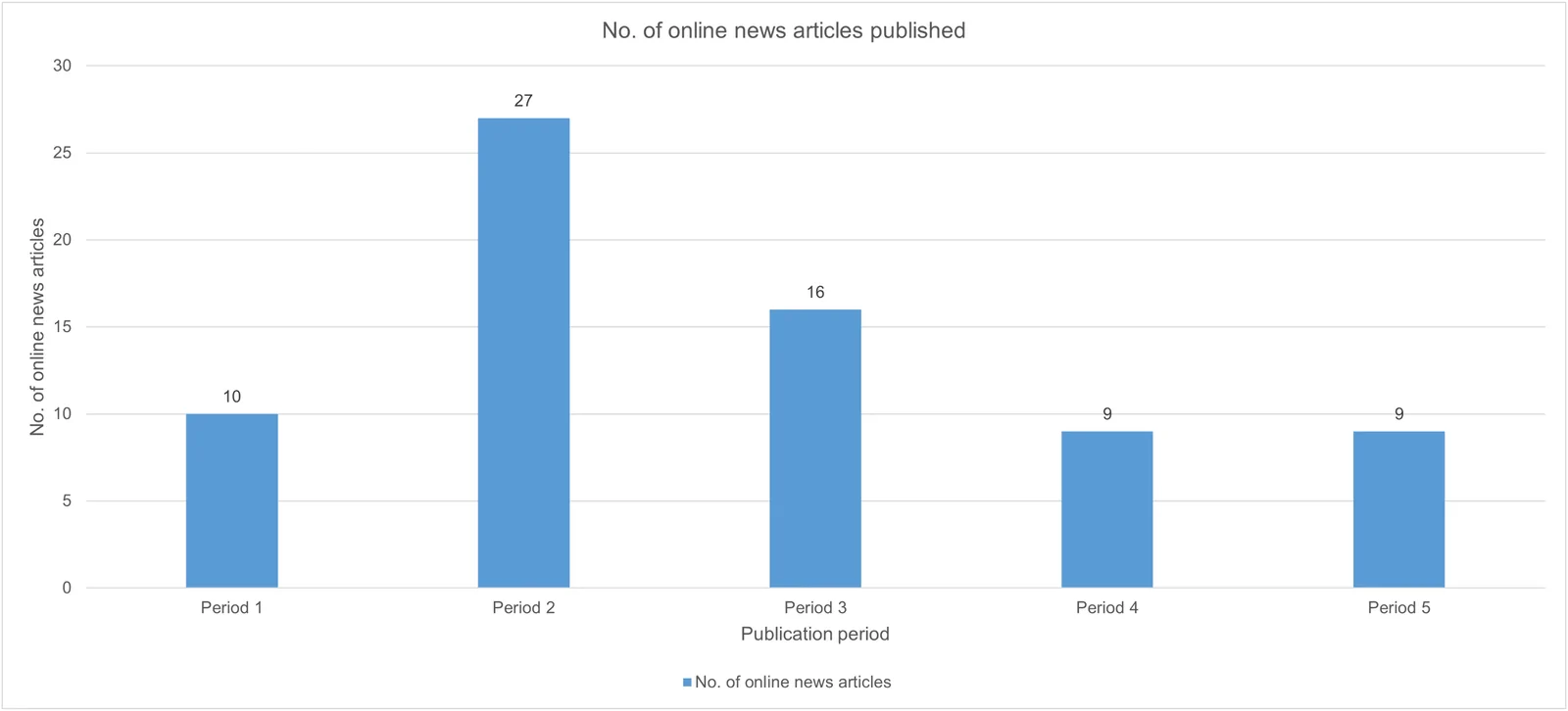
Number of online news articles published by timeframes^ab^ post flood. ^a^ **Period 1:** February/March flood event (28 February 2022 to 29 March 2022); **Period 2:** Within 6 months post-March flood (30 March 2022 to 30 September 2022); **Period 3:** From 6 months post-March flood to 1-year post-February flood (1 October 2022 to 28 February 2023); **Period 4:** From 13 to 18 months post-February flood (1 March 2023 to 31 August 2023); **Period 5:** From 18 to 24 months post-February flood (1 September 2023 to 28 February 2024). ^b^ Period 1 represents the one-month period between the first and second flood event in Lismore in 2022, with Periods 2, 3, 4 and 5 representing subsequent 6-month invervals following the second flood event on 29 March 2022.

Ten articles were published between the first flood event on 28 February 2022 and the second event on 29 March 2022 (Fig 2). Over the next 2 years, the number of online articles published within each 6-month period reduced over time (Fig 2).

### Impact of flooding on Lismore residents

**Fig 3:**
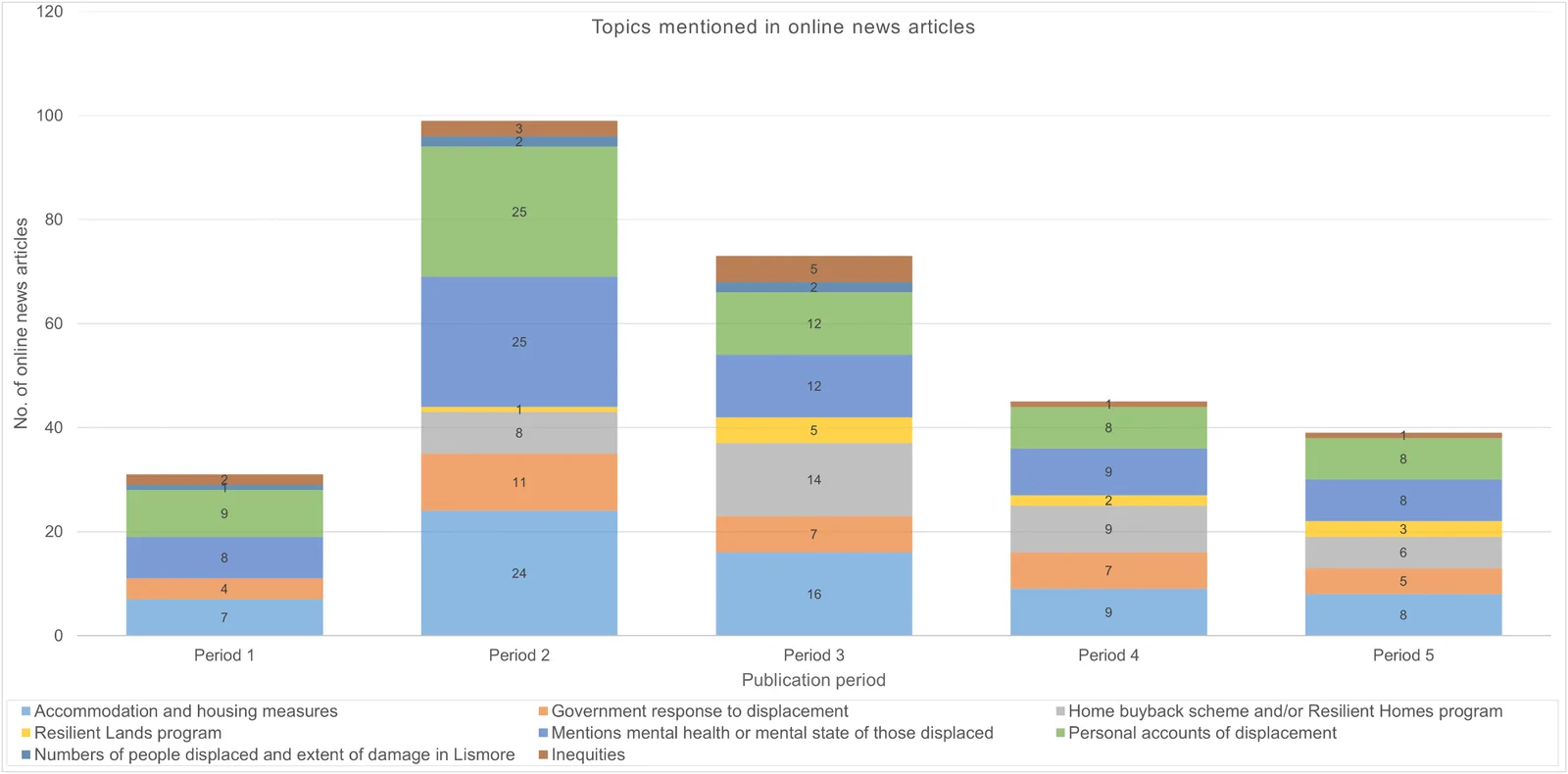
Topics mentioned within online news articles during publication periods^ab^ post flood. ^a^ **Period 1:** February/March flood event (28 February 2022 to 29 March 2022); **Period 2:** Within 6 months post-March flood (30 March 2022 to 30 September 2022); **Period 3:** From 6 months post-March flood to 1-year post-February flood (1 October 2022 to 28 February 2023); **Period 4:** From 13 to 18 months post-February flood (1 March 2023 to 31 August 2023); **Period 5:** From 18 to 24 months post-February flood (1 September 2023 to 28 February 2024). ^b^ more than one topic may have been covered within articles, therefore the sum of the number of articles per topic does not equal total number of articles published per time period.

Only five articles reported on the numbers of people displaced and extent of damage to homes in Lismore, all of which were published within the first year post-flood (Fig 3, Table A in S2 File). These five articles primarily focused on damage to homes, with 4 articles reporting varying statistics ranging from “more than 600 homes” [34] to “over 2,000 homes have been condemned in Lismore region” [35]. One article from August 2022 stated “more than 400 of [people still in emergency accommodation] were from the Lismore area” [36]. No further estimates of displacement in Lismore were reported in online news articles.

### Accommodation and housing measures

Across all time periods, news articles consistently reported on accommodation and housing measures utilised by displaced people (n = 64) (Fig 3, Table A in S2 File). Most reporting focused on government responses (n = 34), particularly the NSW State Government’s Resilient Homes program [37] (n = 37) and Resilient Lands program [38] (n = 11) (Fig 3, Table A in S2 File). Articles reporting on these programs were more prevalent in Periods 3, 4 and 5, coinciding with their implementation (Fig 3, Table A in S2 File). A significant number of articles included personal opinions or accounts of displacement (n = 62), including experiences accessing government housing and accommodation programs (Fig 3, Table A in S2 File). Personal accounts of accessing government programs and schemes characterised the process as “confusing”, “frustrating” and “haphazard” which caused uncertainty and distress [39,40].

Many also expressed confusion regarding the Resilient Lands program which had been intended to increase availability of affordable land and housing located off the flood-plain to allow for land swaps and relocation. It was reported that affordable housing under the Resilient Lands program would not be available until 2026, and as such, residents would need to continue living in long-term temporary accommodation in the meantime [41]. Relocation of homes was reported to be complicated and financially draining, requiring residents to secure land using personal finances before being able to access government funds [42]. Twenty-five articles included mention of people selling their homes on the open market, often at a significant financial loss, as they were either unable or unwilling to wait until a formal government response was made (Table 2).

**Table 2:** Accommodation and housing types accessed^a^, sorted by intended timeframes^b^ of use for accommodation solution (emergency, short-term, long-term temporary and long-term ongoing).

	Emergency	Short-term	Long-term temporary	Long-term ongoing
	Within 2 weeks of flood	Between 2 weeks to 6 months post-flood	Greater than 6 months post-flood, not intended as ongoing solution	Greater than 6 months post-flood, intended as ongoing solution
**Accommodation/housing solution**				
Evacuation centre	11			
Staying with friends	3	11	3	
Staying with family		7	5	1
Staying with other community members	3			
Residing in car	2		2	
Short-stay accommodation (e.g., Airbnb, motels)	1	5	1	
Returning to live in unrepaired flood-damaged home		10	11	
Couch-surfing		7		
Tents		5	5	
Repurposed recreation camps		2		
Caravan on own property		5	14	
Caravan Park		1	1	
Caravan (unspecified location)		6	7	
Renting			6	
Temporary “pods” on own property			8	
“Pod” villages (government-funded)			5	
Selling home through Resilient Homes buyback scheme				37
Moving away from pre-flood home				25
Rebuilding with intent to resume residence				20
Relocation of physical home				14
Selling home on open market				7
Land swaps				7

a more than one accommodation and housing measure may have been covered within each article, therefore the number of articles per accommodation and housing type does not equal the total number of articles

b the time periods listed within this table reflect the nature of the accommodation and intended timeframe of its use. This does not preclude some accommodation types being used beyond their intended timeframe

Informal accommodation arrangements were often mentioned across emergency, short-term and long-term temporary housing measures. Staying with friends or family, living in caravans and returning to live in unrepaired, flood-damaged homes were consistently reported as some of the more common informal arrangements across all timeframes (Table 2).

Many reported returning to live in unrepaired and flood-damaged homes due to lack of alternative options. While in some cases, people returned to their damaged homes with the intention to rebuild and resume ongoing residence, others had “nowhere else to go” [43]. Residents reported these homes to be unsafe and potentially hazardous, citing issues such as mould, unstable foundations, and no walls or doors to protect against cold and weather exposure. One article discussed how potentially hazardous chemical substances were carried by floodwaters to South Lismore homes from an asphalt depot [44]. Another article reported potential asbestos exposure in the community [45].

Difficulty accessing alternative housing options was a common theme across news articles. Flooding exacerbated the existing housing crisis in Lismore as flood-related displacement increased demand and flood damage decreased availability of accommodation [46]. Financial constraints were also a major barrier. Due to high demand, prices for rental accommodation were “through the roof” [46]. However, financial capacity of displaced residents decreased due to loss of income and employment, increased cost of living, and house repair expenses [47].

Safety concerns associated with varying post-flood accommodation measures also influenced decision-making. The safety and health of children often drove decision-making, including choice of emergency shelter and potential relocation away from areas with increased crime [35,48]. Fear of future floods was another concern which influenced decision-making regarding long-term ongoing housing.

### Population groups

**Fig 4:**
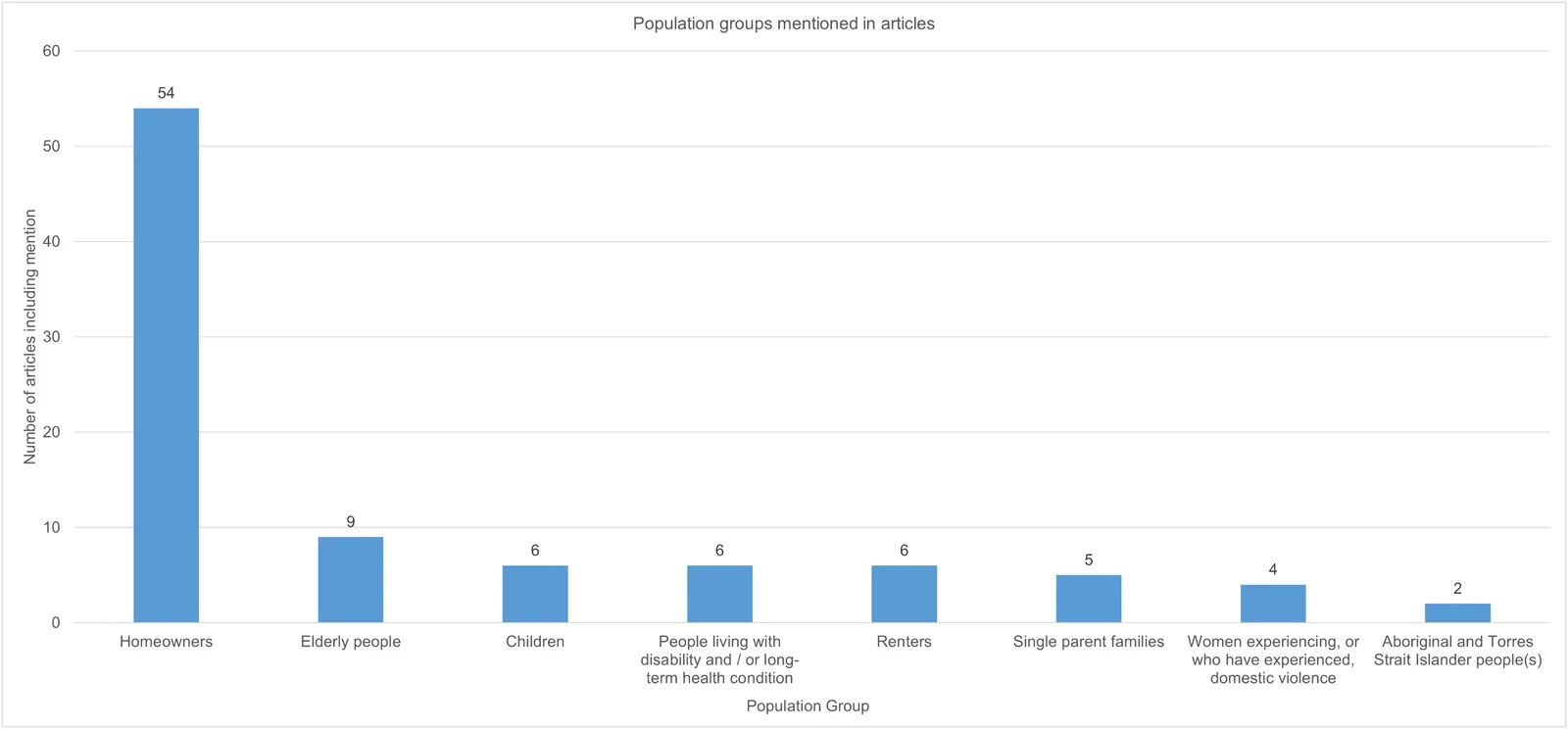
Number of articles with mention of population groups, sorted by frequency mentioned.

A significant proportion of news articles reported on the displacement experiences of homeowners (n = 54) compared to renters (n = 6) (Fig 4, Table B in S2 File). One article attributed this gap in reporting to the majority of remaining residents in Lismore being homeowners, as renters, comparatively, had more latitude and freedom to leave Lismore [49]. In some cases, renters were evicted from their rental homes as their landlords began repairing flood-damaged homes [46,50].

**Fig 5:**
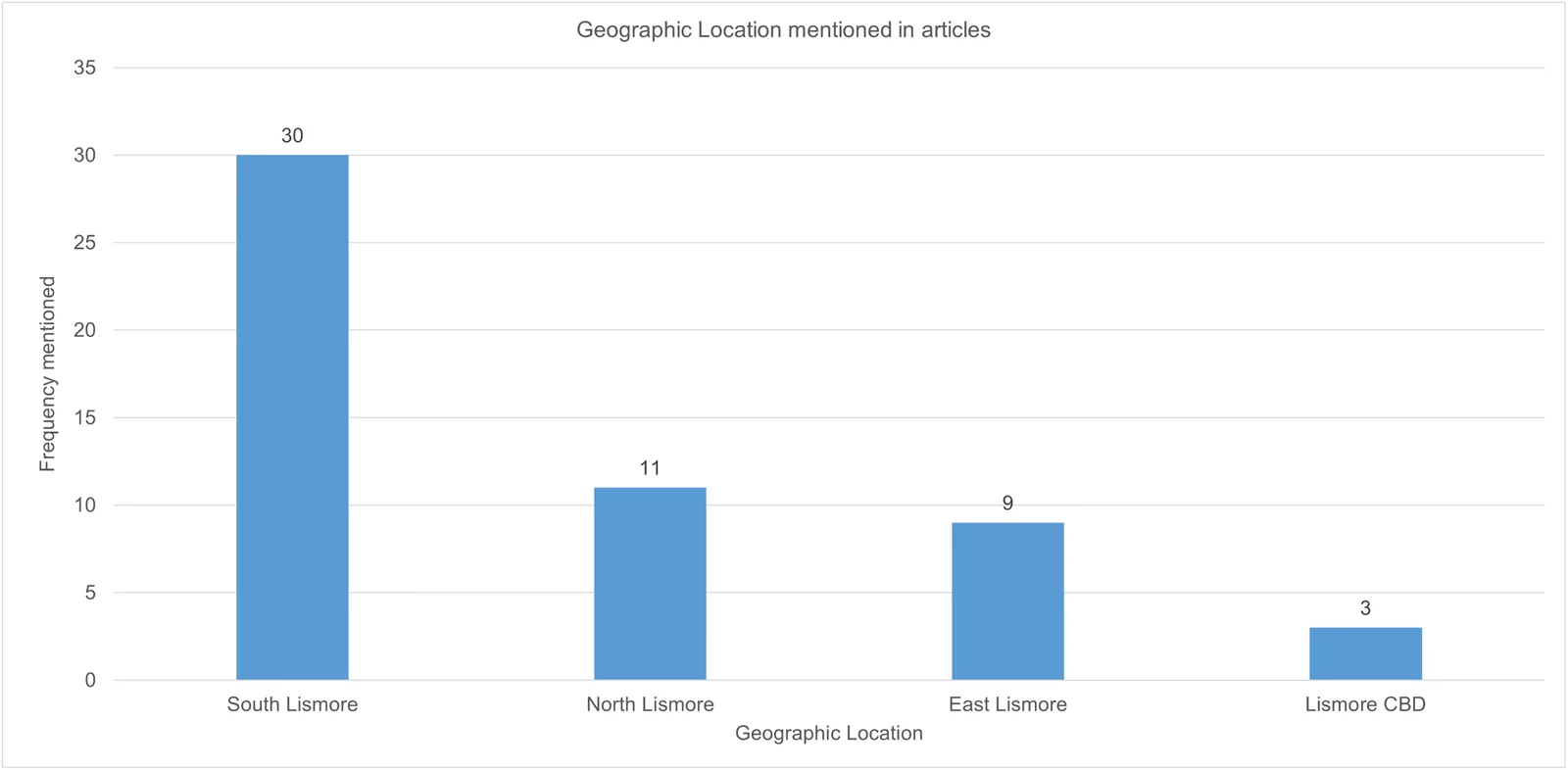
Number of articles with mention of geographic locations, sorted by frequency mentioned.

Reporting on the displacement experiences of specific population groups varied across groups in both frequency (Fig 4, Fig 5, Table B in S2 File) and depth of exploration. Most news articles contained multiple personal accounts from community members across varying population groups. However, relatively limited attention was given to highlighting the inequities between groups (n = 12) (Fig 3, Table A in S2 File).

Nine articles made mention of elderly residents (Fig 4, Table B in S2 File). Poor computer literacy was discussed in two articles as a major barrier to planning and arranging accommodation post-flood for elderly people as many claims processes are completed online [51,52]. Articles also discussed the influence of displacement on physical and mental health in elderly populations. Lack of access to healthcare was a major concern as many elderly people who lived in temporary accommodation were unable to travel to their usual physicians or receive at-home care [51].

Across the six articles which included mention of people with disabilities or health conditions, the primary theme was that there was inadequate support or consideration provided to this group. Articles described “limited forward planning” for how to appropriately support people with disabilities, among other population groups [53].

Children were mentioned in six articles in passing by parents and other community members concerned about the potential influence of flooding and displacement on children’s mental health. Breakdown of family relationships was also discussed, including within parental relationships [43], as well as between parent and child [36].

Four articles discussed the challenges faced by women fleeing domestic violence. First, articles listed concerns that evacuation centres were not equipped to protect women fleeing from their abusers [53]. Further concerns raised within news articles included the risk that women would be forced to stay with abusive partners to avoid homelessness due to limited availability of accommodation [43].

Two articles discussed the experiences of Aboriginal and Torres Strait Islander peoples (Fig 4A, Table B in S2 File). In both articles, the positive influence of community belonging and connection in recovery and rebuilding was explored [54,55]. Both articles also discussed the need for inclusion of Aboriginal and Torres Strait Islander peoples’ perspectives in flood planning and recovery efforts, particularly given their understanding of displacement and connection to Country [54,55].

### Mental health

**Fig 6:**
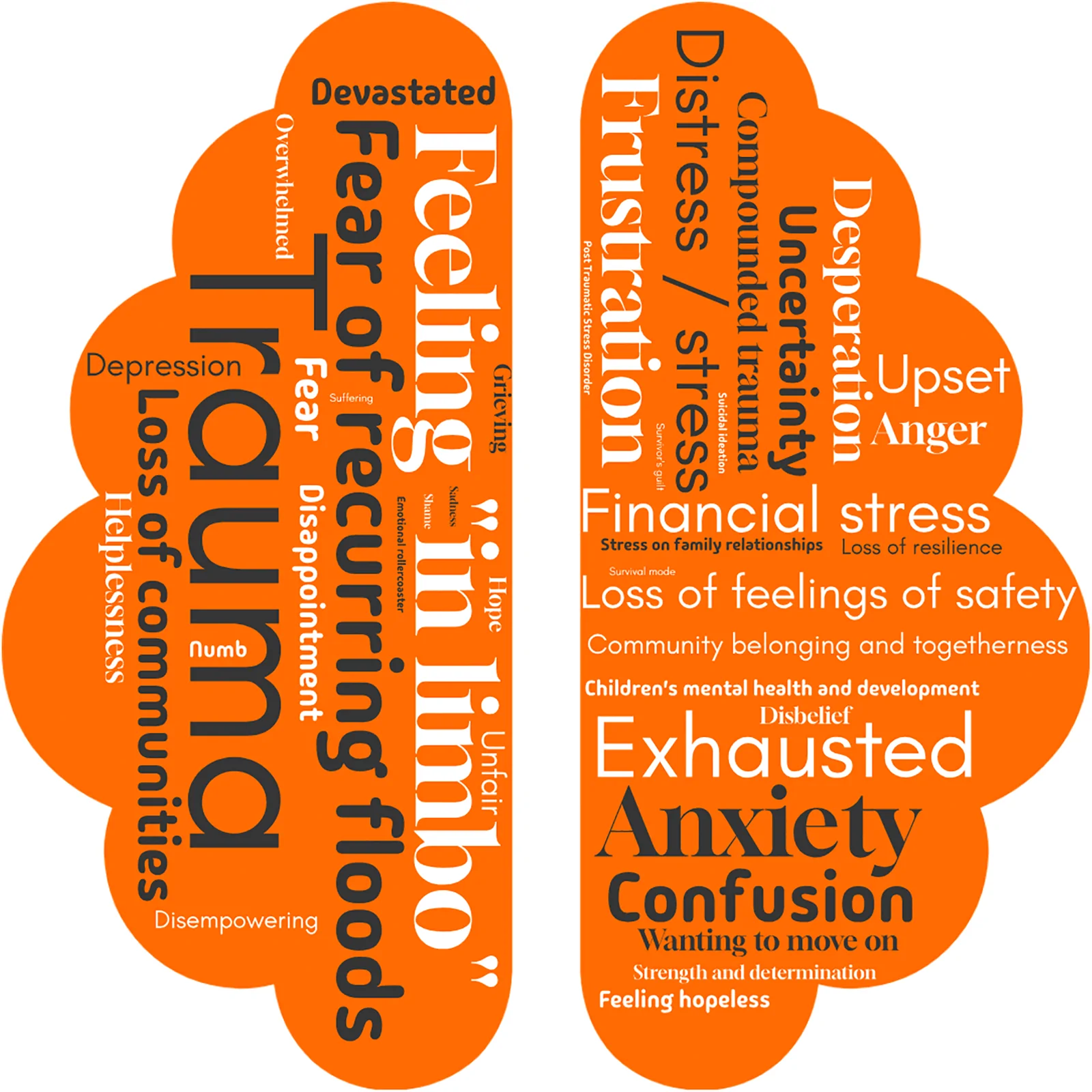
Word cloud illustrating frequently used descriptors of emotional and mental states in online news articles.

Mentions of mental health and mental state were consistently raised in news articles across all time periods published over the two year time period (Fig 3, Table A in S2 File). Overall, ‘traumatised’ was the most common word used within news articles to describe the mental health and emotional states of displaced residents of Lismore (n = 30) (Fig 6, Table C in S2 File). Residents expressed that their trauma was often compounded by other factors such as loss of feelings of safety, financial stress, feeling ‘stuck’ or ‘in limbo’ and difficulties accessing government support. One article described changes in attitudes amongst community members after the second flood in March 2022 where, following the first flood, “people were quite positive” however, “there will be an awful lot of people that decide maybe enough’s enough” following the second flood [47].

News articles reported financial stress (n = 18) was a compounding risk factor for poor mental health among displaced Lismore residents. Many experienced loss of employment and income as businesses and jobs were affected by the floods. Additionally, alongside costly home repairs, it was reported that many homeowners were required to continue paying for mortgages and bills despite their homes being uninhabitable.

Feeling ‘in limbo’ (n = 17) due to uncertainty (n = 11) and lack of timely communication from government organisations was another commonly discussed risk factor for adverse mental health within news articles. Most articles included opinions from community members that the official government response was slow, with many displaced residents resorting to informal accommodation and housing as a result. Residents reportedly felt disempowered and struggled to plan their futures due to limited information regarding possible choices. Furthermore, these long processes were further complicated by loss of identity documents and lack of access to computers and the internet. Many found the processes to be “re-traumatising” [56] and a major barrier to recovery.

As a result, Lismore residents appeared to have more faith and reliance in volunteers and community-led organisations such as Resilient Lismore [57]. Community belonging and togetherness was discussed in eight articles as a protective factor for mental health. Conversely, loss of community (n = 12), including loss of heritage and cultural identity, were listed as risk factors for adverse mental health outcomes.

## Discussion

Online news articles documented experiences of displacement in Lismore following the 2022 floods, including types of accommodation accessed. Decision-making of displaced residents regarding housing and accommodation was typically informed by financial means, availability of housing and accommodation options, and beliefs regarding relative safety of potential options. The experiences of homeowners and opinions regarding the government response to displacement was a major topic of focus within online news reporting, with many articles reporting distress and frustration in relation to poor communication, and confusing and slow bureaucratic processes. There were significantly less articles reporting on the experiences of renters and other population groups compared to homeowners which limited our ability to assess the influence of displacement on these underserved populations. Approximately 86% of articles included at least one reference to the emotional or mental state of displaced residents with ‘traumatised’ being the most used term overall, reflecting the significant influence of displacement on mental health.

From our analysis, informal housing and accommodation arrangements, such as staying with friends and family, returning to reside in flood-damaged homes, and living in caravans, tents and cars were common post-flood solutions reported by displaced residents within Lismore. Informal housing arrangements with family, friends or community members appears to be an important component of the experience of displacement for many which appears to have been recognised in some official publications. For example, a report from Northern Rivers Community Foundation [58] acknowledging widespread ‘inappropriate’ housing situations including people living in cars, couch-surfing, mouldy environments and houses without basic facilities. Furthermore, recommendations from Living Lab Northern Rivers [59] regarding increasing long-term resilience of disaster-affected areas highlight the need for government responses to focus on utilising existing social networks in communities and enabling resident-led solutions.

Our findings also suggest formal accommodation and housing measures, such as evacuation centres, and the Resilient Homes and Resilient Lands programs did not adequately meet the needs of most of the Lismore community. Displaced residents reported poor availability and accessibility, insufficient consideration of the specific needs of population groups, and delayed and flawed implementation of formal programs. This reflects findings from the NSW Auditor-General [60] regarding the post-flood housing response. These challenges are commonly experienced in post-disaster government responses globally [61].

Across all population groups, including renters who have insecure housing tenures, older people, people with disabilities, and Aboriginal and Torres Strait Islander peoples, news reporting coverage did not reflect population proportions [28]. For example, in 2021, 25.7% of the Lismore city population were renting [28], however only 9% of online news articles included any mention of the displacement experiences of renters post-flood. In a report from Living Lab Northern Rivers [59], some local stakeholders in the Lismore region reported concerns regarding inequitable access to housing assistance, with renters reported as being ineligible for support. However, these challenges were rarely discussed in news reporting, with higher focus placed on homeowners, particularly in regard to concerns and challenges associated with accessing the Resilient Homes and Resilient Lands programs.

Furthermore, there was limited news reporting on challenges experienced by various population groups, despite research indicating they are disproportionately affected by climate-related disasters [62]. For example, we found no news articles discussing difficulties faced by rough sleepers, residents who are not Australian citizens and people from the LGBTQIA+ community, despite these populations being highlighted by local stakeholders as being particularly vulnerable to flood displacement [58]. Racism and discrimination against Aboriginal and Torres Strait Islander peoples was also highlighted by local stakeholders as a significant concern [58,59], however there was little exploration of this within news reporting. Challenges faced by older people, women experiencing domestic violence and people with disabilities also did not have high levels of news coverage. However, we found news reporting did accurately reflect views of local stakeholders regarding the inappropriateness of various housing measures for these populations [58,59]. Additionally, news reporting of experiences of older people and people with chronic illness often highlighted the negative health impacts of flood displacement, due in part to disruptions in healthcare and treatment, which is consistent with broader research [62].

Unequal coverage and limited exploration into displacement experiences of specific population groups in news reporting, as found in our study, reflects known issues regarding the poor visibility of these population groups within disaster planning and the broader societal context [62].

Our findings showed there was some consideration of flood-driven exacerbation of socioeconomic and health inequities in news reporting, which reflects concerns raised by local stakeholders [58,59] and climate research [16]. This included concerns that low-income households would remain in high-risk flood zones due to societal and financial constraints. Previous research, such as Peacock et al [16], found low-income neighbourhoods in the United States with more physical and social vulnerability experienced higher losses and damage, and slower post-disaster housing recovery. We found this to be consistent with our findings, where disaster recovery efforts in Lismore have been prolonged, with many residents still without stable housing more than three years post-flood. News reporting also describe residents forced to return to flood-damaged homes being exposed to numerous environmental health hazards, including pollutants carried by flood-waters and mould, which can lead to poor physical health and illness, resulting in widening health inequities. This is consisted with other research regarding the negative health impacts of flood displacement [63].

We found news reporting to be consistent with broader research that indicates the experience of displacement and the process of securing accommodation can be distressing, and increases risk for the development of mental health disorders such as PTSD, depression and anxiety in those who have been displaced [64]. We also found evidence to support other findings that flood survivors can be re-traumatised while attempting to access formal planning, housing and accommodation assistance [63]. Our findings demonstrate that uncertainty and feeling ‘in limbo’ are major contributing factors to poor mental health, which supports findings from Blake et al [14] that having agency, control and choice with housing post-disaster results in better long-term health and wellbeing outcomes.

### Strengths and limitations

Limitations of this study include that it only examined online news articles reporting on displacement and post-disaster housing within the Lismore LGA. Therefore, some of these findings may not be generalisable to other contexts.

Due to limited availability of relevant articles through common newspaper aggregate databases, this study was limited to online news articles, identified through Google News, with free access through unpaid membership or through the University of Sydney Library. It is possible that the selected articles may be a non-representative or biased sample of news reporting as some research suggests Google News is more likely to provide results from national news outlets than local news outlets [65]. Furthermore, the nature of news reporting results in a limited perspective due to potential media bias in reporting. Information and stories which are considered newsworthy may not reflect experiences of displacement across the whole community or may not be important or relevant to communities in their day-to-day context.

To our knowledge, this is the only study to utilise online news articles to examine experiences of flood-related displacement and its influence on mental health. While the information that can be gathered from newspapers is limited in scope, it nonetheless provides a record of issues of interest at various times over the two year period after the major flood events of February 2022 [66]. News reporting also helps provide a general overview of priorities and views held within communities as issues that attracted journalist interest will, to some extent, reflect community concerns [66]. Journalists also typically collect stories and opinions from affected people close in time to occurrence of an event.

Authors RM, JB, and RB are embedded researchers at the University Centre for Rural Health in Lismore, which was at the epicentre of the flooding events and this embeddedness may have influenced their approach to the study and their perspectives on the data.

### Implications

This study provides a general understanding and overview of the experiences and opinions of people who were displaced in Lismore following the 2022 NSW floods as reported in online news articles. These findings support recommendations from the 2022 NSW Flood Inquiry [67] regarding possible improvements to service delivery and implementation of future policies and initiatives, including clear communication, timely responses, and the importance of community participation in flood response and recovery planning. The findings from this study support the need for enhanced disaster recovery planning for building temporary housing and transitioning to long-term housing to ensure safety and protection of displaced peoples, and using a needs-based approach to ensure policies are context-appropriate and address the housing challenges faced by all groups within communities [12,61,67,68]. These findings reiterate the need for specific policy and legislative mechanisms for governing internal displacement in Australia [69], especially within the context of increased likelihood of large scale disasters.

Consideration of the challenges faced by population groups such as elderly people, people with disabilities or chronic illness, Aboriginal and Torres Strait Islander peoples, single parent families, children and low-income earners is particularly important as displacement can otherwise result in furthering disadvantage and widening inequities within communities. Early involvement of communities in flood recovery planning is recommended by the United Nations Office for Disaster Risk Reduction [70] as it helps to empower communities and leads to better success in recovery initiatives. As such, community participatory research and consultation can be important protective factors in reducing flood inequities [12,23,62].

## Conclusion

In summary, it is likely extreme weather events and flooding will be further exacerbated by climate change and therefore disaster-related housing displacement and the associated health impacts will be an increasing challenge [1]. Given our findings indicate an association between displacement and poor mental health outcomes, trauma-informed communications should be at the heart of housing displacement policy and programs. Our findings support the need for collaboration between communities and governments in flood recovery planning to ensure policies and programs are context-appropriate and relevant, and to empower and return agency to displaced people and communities. In general, our findings further support the need for internal displacement-specific policy and legislative mechanisms in Australia in line with international standards.

Our findings demonstrate how news articles can provide a novel and unique lens from which useful information can be gathered on contemporary social issues. Further research and analysis of news articles from local news outlets in the broader flood affected Northern Rivers region (seven LGAs) within which Lismore is situated may provide more relevant and specific information regarding experiences of displacement across the general population and for various population groups. Analysis of the level of news coverage and tone of news articles discussing the experiences of population groups may be helpful in assessing societal and media attitudes towards inequities between various population groups. We have found that informal housing plays a significant role in post-flood accommodation options, indicating the need for improved understanding of this modality within research and policy domains.

## Supporting information

S1 File List of articles used in analysis. (DOCX)

S2 File Contains Table A (Topics covered within online news articles, categorised by date of publication), Table B (Population groups and geographic locations, frequency mentioned) and Table C (Emotional and mental states identified in news articles, in descending order of frequency). (DOCX)
