# Supplementary material for: Housing displacement experiences following major flooding in regional Australia: A qualitative study of online news articles
Source: PLoS One. 2026 Sep 9;21(9):e0357805. doi: 10.1371/journal.pone.0357805 (PMC13557363; doi:10.1371/journal.pone.0357805)
Supplement: Supplementary file 1 [file pone.0357805.s003.pdf]

Housing displacement experiences following major flooding in regional Australia: a qualitative study of online news articles

The study investigated the nature and scope of media reports on the experience of people displaced from their homes and the influence of access to accommodation on physical and mental health and well-being after a large flood in the Australian region.

The study has clear objectives, an appropriate methodology, and design with sufficient data saturation. There was a balanced discussion and clear findings. It has research value with appropriate contributions. The structure was consistent with adequate integration of the literature.

Nevertheless, I think the authors could use other methods of presenting data other than the use of tables. This, I believe, will enhance the quality of the paper. Kindly note that Figure 2 is missing from the submission.

In general, the paper can be published if minor corrections are effected.
