# Supplementary material for: Housing displacement experiences following major flooding in regional Australia: A qualitative study of online news articles
Source: PLoS One. 2026 Sep 9;21(9):e0357805. doi: 10.1371/journal.pone.0357805 (PMC13557363; doi:10.1371/journal.pone.0357805)
Supplement: Supplementary file 2 [file pone.0357805.s005.pdf]

Housing displacement experiences following major flooding in regional Australia: a qualitative study of online news articles

The article addresses an important social challenge of housing displacement after flooding. Health and equity implications were drawn from a qualitative analysis of news articles and the gaps were equally highlighted. There is coherency in the narrative, but for consistency and clarity, the areas to be strengthened include:

1. **Tables**
   In Table 1 - check whether 'World Socialist Wide Web' is World Socialist Website'. Table 2 - the footnote can be moved into the caption for readability. Bolden the highest values in each row to highlight trends. It will be more appropriate to use the same time interval (six months). Table 3 - clarify overlaps and ensure consistency e.g., caravans, Pod villages, and Temporary pods. Table 4 – vulnerable populations (Indigenous people, disabled, single parents, and elderly) can be grouped together under discussion for emphasis. Table 5 - Mental health descriptors can be put together into categories to aid interpretation.
2. **Figures**
   Use 6-month intervals in Figure 2
3. **Overall Recommendation:**
   Shorten the abstract sentences and concentrate on the novelty of using news articles as a qualitative source. In the discussion, expand on the policy implications, that is, how government can better integrate informal housing arrangements into disaster planning. Point out the limitations of the study, such as potential bias of the media reporting. Let the conclusion be directed more to policy makers and researchers as a wake-up call to action.

All in all, the paper is solid and publishable. The authors should make the adjustments with respect to the tables and figure. Expanded discussion on vulnerable groups and policy implications will strengthen the relevance of the article.
