# Supplementary material for: Housing displacement experiences following major flooding in regional Australia: A qualitative study of online news articles
Source: PLoS One. 2026 Sep 9;21(9):e0357805. doi: 10.1371/journal.pone.0357805 (PMC13557363; doi:10.1371/journal.pone.0357805)
Supplement: Supplementary file 3 [file pone.0357805.s001.docx]

**S1 Table. List of online articles included in analysis, sorted by date of publication**

| **Publication Date** | **Author(s)** | **Title of article** | **News outlet** |
| --- | --- | --- | --- |
| 28/02/2022 | Eden Gillespie Dom Vukovic | Flood victims have 'lost everything' in northern NSW disaster | SBS News |
| 4/03/2022 | Bhiamie Williamson | Like many disasters in Australia, Aboriginal people are over-represented and under-resourced in the NSW floods | The Conversation |
| 5/03/2022 | Elias Visontay | Perrottet defends NSW flood response amid Lismore anger as QLD death toll rises | The Guardian |
| 6/03/2022 | Christine Tondorf | ‘Worse than 2017’: Lismore faces mammoth rebuild after flood as community inundated by loss | The Guardian |
| 6/03/2022 | Heath Gilmore | ‘This wasn’t a flood, it was a demolition’: How can Lismore survive? | Sydney Morning Herald |
| 10/03/2022 | Cait Kelly | Pressure grows on Airbnb to permit long-term rentals as NSW floods worsen housing crisis | The Guardian |
| 14/03/2022 | Antonia Settle | After the floods, the distressing but necessary case for managed retreat | The Conversation |
| 17/03/2022 | Elias Visontay | ‘It’s stuff you can’t replace’: experts fear priceless local history will be thrown out in NSW floods cleanup | The Guardian |
| 18/03/2022 | Elias Visontay Tamsin Rose | Motor homes for flood-affected Lismore residents empty while more temporary housing yet to arrive | The Guardian |
| 20/03/2022 | Elias Visontay | Flood-affected Lismore residents with nowhere to go return to homes deemed uninhabitable | The Guardian |
| 30/03/2022 | Caitlin Cassidy | Northern NSW floods: evacuation order reissued for Lismore CBD as levee expected to be breached | The Guardian |
| 30/03/2022 | Christine Tondorf Cait Kelly Conal Hanna | Lismore and Byron Bay flooded as weather system tracks south bringing more evacuations | The Guardian |
| 31/03/2022 | David Lowe | Lismore floods again, levee overtopped – The Echo | Echonetdaily |
| 31/03/2022 | Susan Chenery | ‘There’s a cracking’: residents are broken in Lismore after the NSW town is flooded again | The Guardian |
| 2/04/2022 | Joe Hinchliffe | Adrift in a parallel universe: Lismore struggles to find itself after another flood | The Guardian |
| 9/04/2022 | susan chenery | Land swaps, relocations or rebuilds: Lismore community grapples with its future | The Guardian |
| 13/04/2022 | Ginger Gorman | Lismore floods: 'We escaped with our lives' | BroadAgenda |
| 4/05/2022 | Christine Tondorf | Trauma, disbelief and plenty of ideas shared at NSW flood inquiry hearing in Lismore | The Guardian |
| 14/05/2022 | Nick Parmeter Emma Rennie | Flood-hit Northern Rivers legal practices struggling as system grinds to a halt amid intense demand | ABC News |
| 28/05/2022 | Bronwyn Herbert | Months on from devastating Lismore floods, 1,300 people are still unable to return home | ABC News |
| 1/06/2022 | Christine Tondorf | Fights broke out as top-down approach to Lismore flood response failed, inquiry told | The Guardian |
| 4/06/2022 | Tamsin Rose | ‘We knew straight away’: Lismore family reunited with lost cat Kimba 13 weeks after their house flooded | The Guardian |
| 12/06/2022 | Harriet Tatham | Lismore residents whose homes were covered in oil from Boral asphalt depot after floods left with daunting clean up | ABC News |
| 6/07/2022 | Ella Archibald-Binge | Housing is the 'missing piece' in flood recovery, as thousands continue living in tents and mouldy homes | ABC News |
| 11/07/2022 | Susan Chenery | Homes of last resort: the pods housing a NSW community ravaged by floods | The Guardian |
| 12/07/2022 | Harriet Tatham | 'Catastrophic' impact of NSW floods sees many elderly people going into residential care earlier than expected | ABC News |
| 16/07/2022 | Bruce Mackenzie | Housing buyback details for Northern Rivers flood zone expected in weeks | ABC News |
| 27/07/2022 | Caitlin Cassidy | Lismore residents with flood-damaged homes still received bills from energy companies | The Guardian |
| 1/08/2022 | John van Tiggelen | Rethinking Lismore in the new era of floods | The Monthly |
| 12/08/2022 | Bronwyn Herbert  Bruce Mackenzie  Hannah Ross | Flood-ravaged communities split amid backlash to temporary pod housing plan | ABC News |
| 14/08/2022 | Olivia Grace Curran  Gracie Richter | 'Live here or be homeless': Lismore residents want the option to relocate their homes after devastating floods | 9News |
| 21/08/2022 | Tamsin Rose | ‘A family now’: the volunteers helping NSW flood victims six months on | The Guardian |
| 30/08/2022 | Bronwyn Herbert  Hannah Ross | Lismore residents and business reflect on six months on from catastrophic floods | ABC News |
| 24/09/2022 | Aidan Devine | Homes ravaged in 2022 NSW floods for sale at prices that hark back to 2005 | The Daily Telegraph |
| 27/09/2022 | Bronwyn Herbert | Lismore Council estimates $500 million spend on retreat plan for flood-affected homes | ABC News |
| 27/09/2022 | Kate Stroud | I’ve moved back home to Lismore just in time for the third La Niña. The sound of rain brings dread | The Guardian |
| 27/09/2022 | Leah White | Flood-affected Northern Rivers residents say trauma compounded by lack of government plans | ABC News |
| 27/10/2022 | Lauren Pezet  Bruce Mackenzie | Northern Rivers flood buyback scheme offered to those with destroyed and damaged homes | ABC News |
| 28/10/2022 | Tamsin Rose | Lismore residents can take their homes with them under $800m buyback program | The Guardian |
| 28/10/2022 | Bruce Mackenzie  Hannah Ross | Northern Rivers flood buyback scheme offers residents fresh start, but some fear losing community | ABC News |
| 28/10/2022 | Richard Wood | NSW government to buy flood-hit homes in $800 million buyback scheme | 9News |
| 29/10/2022 | Hannah Ross  Lucy Thackray | Home buyback scheme for high-risk flood zones in the NSW Northern Rivers explained | ABC News |
| 24/11/2022 | Bruce Mackenzie | Lismore flood victims in grip of housing crisis still as report urges change to cope with disasters | ABC News |
| 9/02/2023 | Leah White | Insurance crisis deepens for homes, business and local councils in flood prone areas of northern NSW | ABC News |
| 20/02/2023 | Susan Chenery | The never-ending fallout of the northern rivers floods: ‘People are just worn down’ | The Guardian |
| 20/02/2023 | Mike Head | One year on, Australian flood victims still left abandoned | World Socialist Web Site |
| 22/02/2023 | Bronwyn Herbert  Madeleine Cross | NSW Resilient Homes buybacks begin in Northern Rivers but anxious residents feel 'no certainty' | ABC News |
| 23/02/2023 | Mark Saunokonoko | 'Nobody's come home': Flood limbo pushes Lismore family to the brink | 9 News |
| 27/02/2023 | Emma Rennie  Leah White  Sharon Gordon | In harm's way | ABC News |
| 27/02/2023 | Rebecca Masters | Lismore floods: The community that saved itself is still in a 'long, hard slog' to recovery | 9 News |
| 27/02/2023 | Gianni Francis  Catherine Piltz  Alexi Demetriadi | Northern NSW flood recovery at a crawl year on from record natural disasters in February-March 2022 | Lismore Northern Star |
| 28/02/2023 | Stefan Armbruster  Amy Hall | A year on from the devastating Lismore flood, Greg and Vicki are still living in a caravan | SBS News |
| 27/04/2023 | Michael Head | Anger over sham “buy-back” scheme deepens in Australian flood-hit region | World Socialist Web Site |
| 22/05/2023 | Dr Glenn Morrison | As Lismore slowly repairs it's preparing for higher floods under climate change | Echonetdaily |
| 2/06/2023 | Gianni Francis  Catherine Piltz | Northern Rivers flood survivors complain of being treated like ‘garbage’ as buybacks program falls behind schedule | Lismore Northern Star |
| 13/06/2023 | Catherine Naylor | Almost 80 per cent of flood victims denied ‘pathway to safety’ | Sydney Morning Herald |
| 20/06/2023 | Gianni Francis  Catherine Piltz | ‘Cut off and isolated’: South Lismore low priority for NRRC despite flood deaths | Lismore Northern Star |
| 25/06/2023 | Joe Hinchliffe | 'We have to get out': despair returns to Lismore for those left out by flood buyback program | The Guardian |
| 28/06/2023 | Catherine Naylor | Marge died in a flooded home, but her house is still not considered dangerous | Sydney Morning Herald |
| 29/06/2023 | Leah White | Two homes where people died in Lismore's February 2022 flood not eligible for government buybacks | ABC News |
| 5/07/2023 | Leah White  Emma Rennie  Julie Ray | Lismore residents face 'cruel' buyback rejection while council considers banning new houses | ABC News |
| 30/07/2023 | David Maurice Smith | Washed away: the struggle to make sense of the northern rivers floods, the largest in modern Australian history | The Guardian |
| 30/09/2023 | Emma Rennie | Lismore residents rally for government improvements to buyback scheme home relocations | ABC News |
| 25/10/2023 | Adam Harvey | Leone's neighbours have been offered buybacks after the Lismore floods. She's not eligible for anything | ABC News |
| 24/11/2023 | Catherine Piltz | South Lismore flood battler Roger Manby, 86, finally home 18 months after 2022 floods destroyed his home | Lismore Northern Star |
| 15/12/2023 | Sam Stolz | Northern NSW flood recovery: Community weighs in on NSW government-led Resilient Homes Program after 2022 disasters | Lismore Northern Star |
| 6/01/2024 | Cindy Wockner | Inquiry submissions reveal frustration with insurance industry over handling of 2022 floods | ABC News |
| 16/01/2024 | Catherine Piltz | Lismore original cottage relocated after flooding | Lismore Northern Star |
| 9/02/2024 | Emma Rennie  Bruce Mackenzie | NSW Premier unveils Resilient Lands Program to frustrated 2022 Lismore flood survivors | ABC News |
| 22/02/2024 | Bruce Mackenzie | Auditor-general's flood report finds NSW had 'no plan' to house 2022 Lismore survivors | ABC News |
| 28/02/2024 | Emma Rennie | Rebuilding continues after floods as Resilient Homes buyback program leaves Lismore with vacant houses | ABC News |
