# Supplementary material for: Housing displacement experiences following major flooding in regional Australia: A qualitative study of online news articles
Source: PLoS One. 2026 Sep 9;21(9):e0357805. doi: 10.1371/journal.pone.0357805 (PMC13557363; doi:10.1371/journal.pone.0357805)
Supplement: Supplementary file 4 [file pone.0357805.s002.docx]

# Supporting Information File 2

# Housing displacement experiences following major flooding in regional Australia: a qualitative study of online news articles

**Authors & Affiliations:**

Jessica Chen^1^, Rebecca McNaught^1,2^, Jodie Bailie^2,3^, Kanchana Ekanayake^4,^ Ross Bailie^1^

^1^ School of Public Health, The University of Sydney

^2^ University Centre for Rural Health, The University of Sydney

^3^ Centre for Disability Research and Policy, The University of Sydney

^4^ University of Sydney Library, The University of Sydney

## Corresponding author:

Dr. Rebecca McNaught^1,2^
Email: [Rebecca.mcnaught@sydney.edu.au](mailto:Rebecca.mcnaught@sydney.edu.au)

**This file includes:**

**Table A.** Topics covered within online news articles, categorised by date of publication

**Table B.** Population groups and geographic locations, frequency mentioned

**Table C.** Emotional and mental states identified in news articles, in descending order of frequency

**Table A. Topics covered within online news article, categorised by date of publication**

|  | Period 1^b^ | Period 2 | Period 3 | Period 4 | Period 5 |
| --- | --- | --- | --- | --- | --- |
|  | 28 Feb 2022 – 29 Mar 2022 | Within 6 months post-March flood (30 March 2022 to 30 September 2022) | From 6 months post-March flood to 1-year post-February flood (1 October 2022 to 28 February 2023) | From 13 to 18 months post-February flood (1 March 2023 to 31 August 2023) | From 18 to 24 months post-February flood (1 September 2023 to 28 February 2024) |
| **Topics covered** |  |  |  |  |  |
| Accommodation and housing measures | 7 | 24 | 16 | 9 | 8 |
| Government response to displacement | 4 | 11 | 7 | 7 | 5 |
| Home buyback scheme and/or Resilient Homes program | 0 | 8 | 14 | 9 | 6 |
| Resilient Lands program | 0 | 1 | 5 | 2 | 3 |
| Mentions mental health or mental state of those displaced | 8 | 25 | 12 | 9 | 8 |
| Personal accounts of displacement | 9 | 25 | 12 | 8 | 8 |
| Numbers of people displaced and extent of damage in Lismore | 1 | 2 | 2 | 0 | 0 |
| Inequities | 2 | 3 | 5 | 1 | 1 |
| **Total number of articles published per time period^a^** | **10** | **27** | **16** | **9** | **9** |

^a^ more than one topic may have been covered within articles, therefore the sum of the number of articles per topic does not equal total number of articles published per time period

^b^ Period 1 represents the one-month period between the first and second flood event in Lismore in 2022, with Periods 2, 3, 4 and 5 representing subsequent 6-month invervals following the second flood event on 29 March 2022.

**Table B. Population groups and geographic locations, frequency mentioned**

| **Characteristic** | **No. of articles** | **% of total articles** |
| --- | --- | --- |
| **Population groups** |  |  |
| Homeowners | 54 | 76 |
| Elderly people | 9 | 13 |
| Children | 6 | 8 |
| People living with disability and / or long-term health condition | 6 | 8 |
| Renters | 6 | 8 |
| Single parent families | 5 | 7 |
| Women experiencing, or who have experienced, domestic violence | 4 | 6 |
| Aboriginal and Torres Strait Islander people(s) | 2 | 3 |
| **Geographic location** |  |  |
| South Lismore | 30 | 42 |
| North Lismore | 11 | 15 |
| East Lismore | 9 | 13 |
| Lismore CBD | 3 | 4 |

**Table C. Emotional and mental states identified in news articles, in descending order of frequency**

| **Emotional descriptor** | **No. of articles** | **% of total articles** |
| --- | --- | --- |
| Trauma | 30 | 42 |
| Financial stress | 18 | 25 |
| Loss of feelings of safety | 18 | 25 |
| Feeling ‘in limbo’ | 17 | 24 |
| Frustration | 16 | 23 |
| Exhausted | 14 | 20 |
| Fear of recurring floods | 14 | 20 |
| Loss of communities | 12 | 17 |
| Distress / stress | 11 | 15 |
| Uncertainty | 11 | 15 |
| Anxiety | 11 | 15 |
| Confusion | 11 | 15 |
| Devastated | 8 | 11 |
| Community belonging and togetherness | 8 | 11 |
| Compounded trauma | 8 | 11 |
| Desperation | 8 | 11 |
| Wanting to move on | 6 | 8 |
| Children’s mental health and development | 6 | 8 |
| Upset | 5 | 7 |
| Anger | 5 | 7 |
| Feeling hopeless | 5 | 7 |
| Fear | 4 | 6 |
| Strength and determination | 4 | 6 |
| Depression | 3 | 4 |
| Disappointment | 3 | 4 |
| Helplessness | 3 | 4 |
| Unfair | 3 | 4 |
| Disempowering | 3 | 4 |
| Grieving | 2 | 3 |
| Hope | 2 | 3 |
| Loss of resilience | 2 | 3 |
| Overwhelmed | 2 | 3 |
| Stress on family relationships | 2 | 3 |
| Disbelief | 2 | 3 |
| Numb | 2 | 3 |
| Emotional rollercoaster | 1 | 1 |
| Sadness | 1 | 1 |
| Shame | 1 | 1 |
| Suffering | 1 | 1 |
| Suicidal ideation | 1 | 1 |
| Survival mode | 1 | 1 |
| Survivor’s guilt | 1 | 1 |
| Post Traumatic Stress Disorder | 1 | 1 |
