# Supplementary material for: Housing displacement experiences following major flooding in regional Australia: A qualitative study of online news articles
Source: PLoS One. 2026 Sep 9;21(9):e0357805. doi: 10.1371/journal.pone.0357805 (PMC13557363; doi:10.1371/journal.pone.0357805)
Supplement: Supplementary file 5 [file pone.0357805.s006.docx]

## Response to reviewers

**Paper for PLOS ONE**

**Paper:** Housing displacement experiences following major flooding in regional Australia: a qualitative study of online news articles

**Authors:** Jessica Chen, Rebecca McNaught, Jodie Bailie, Kanchana Ekanayake, Ross Bailie

Thank you to the reviewers and editors for their comments. We have addressed these comments in the table below.

| **Reviewer Comment** | **Response** |
| --- | --- |
| Reviewer #1:  The manuscript was well-written, clear and concise, and engaging to read. Having conducted qualitative research with rural communities impacted by flood, I found that the findings presented here were consistent with my own. I feel that the manuscript could be published in its current form but have a minor comment for the authors to consider below.  1. Given that the study was underpinned by a constructivist epistemology, and the study involved a review of online new articles related to flooding in the researchers' own community, how were the researchers’ own experiences of flood/flood-related displacement considered or drawn on throughout the analysis process? How did the researchers’ own experiences influence decisions made during the analysis and their making meaning of the data? It might be useful to including a reflexive statement describing the researchers’ experiences and how these experiences influenced their decision to undertake this research and may shape their interpretations of the data. | Thank you for picking this up. To address your comment, we have added a reflexive statement in the methods section which better states how our analysis and topic selection was influenced by the lived experience of the researchers. |
| Reviewer #2  Housing displacement experiences following major flooding in regional Australia: a qualitative study of online news articles  The study investigated the nature and scope of media reports on the experience of people displaced from their homes and the influence of access to accommodation on physical and mental health and well-being after a large flood in the Australian region.  The study has clear objectives, an appropriate methodology, and design with sufficient data saturation. There was a balanced discussion and clear findings. It has research value with appropriate contributions. The structure was consistent with adequate integration of the literature.  Nevertheless, I think the authors could use other methods of presenting data other than the use of tables. This, I believe, will enhance the quality of the paper. Kindly note that Figure 2 is missing from the submission.  In general, the paper can be published if minor corrections are effected. | Thank you for your feedback. In response, we have made the following changes:   1. Reattached Figure 2 2. Adapted data from Table 2 into Figure 3 (stacked column chart) 3. Adapted data from Table 4 into Figures 4 and 5 (column charts) 4. Adapted data from Table 5 into Word Cloud in Figure 6 5. Moved Tables 2,4 & 5 to Tables A, B & C in Supporting Information File 2 |
| **General Comments** | |
| Please ensure that your manuscript meets PLOS ONE's style requirements, including those for file naming. The PLOS ONE style templates can be found at [https://journals.plos.org/plosone/s/file?id=wjVg/PLOSOne_formatting_sample_main_body.pdf](https://url.au.m.mimecastprotect.com/s/yApiC3QNPBiG7LLJzCDsJSQHrF4?domain=track.editorialmanager.com) and [https://journals.plos.org/plosone/s/file?id=ba62/PLOSOne_formatting_sample_title_authors_affiliations.pdf](https://url.au.m.mimecastprotect.com/s/OhysC4QOPEimYLLQKUVtOS4XAei?domain=track.editorialmanager.com) | We confirm our manuscript complies with PLOS ONE’s style requirements, including file naming conventions, to the best of our knowledge. |
| Your ethics statement should only appear in the Methods section of your manuscript. If your ethics statement is written in any section besides the Methods, please delete it from any other section. | We confirm the ethics statement has been moved to the Methods section of our manuscript. |
| We note that your Data Availability Statement is currently as follows: All relevant data are within the manuscript and its Supporting Information files.  Please confirm at this time whether or not your submission contains all raw data required to replicate the results of your study. Authors must share the “minimal data set” for their submission. PLOS defines the minimal data set to consist of the data required to replicate all study findings reported in the article, as well as related metadata and methods ([https://journals.plos.org/plosone/s/data-availability#loc-minimal-data-set-definition](https://url.au.m.mimecastprotect.com/s/fqVhC5QPXJixgqqQLi8uRSkcxub?domain=journals.plos.org)).  For example, authors should submit the following data:  - The values behind the means, standard deviations and other measures reported; - The values used to build graphs; - The points extracted from images for analysis.  Authors do not need to submit their entire data set if only a portion of the data was used in the reported study.  If your submission does not contain these data, please either upload them as Supporting Information files or deposit them to a stable, public repository and provide us with the relevant URLs, DOIs, or accession numbers. For a list of recommended repositories, please see [https://journals.plos.org/plosone/s/recommended-repositories](https://url.au.m.mimecastprotect.com/s/YZpYC6XQ4Lf1VQQAYUBCqS58IPL?domain=journals.plos.org).  If there are ethical or legal restrictions on sharing a de-identified data set, please explain them in detail (e.g., data contain potentially sensitive information, data are owned by a third-party organization, etc.) and who has imposed them (e.g., an ethics committee). Please also provide contact information for a data access committee, ethics committee, or other institutional body to which data requests may be sent. If data are owned by a third party, please indicate how others may request data access. | We confirm that we have included all the relevant data in our application. Please see S1 File for a full list of the online news articles analysed in this study. |
| Your abstract cannot contain citations. Please only include citations in the body text of the manuscript, and ensure that they remain in ascending numerical order on first mention. | We note our abstract does not contain citations however we have amended our numbering method to avoid any confusion. |
| [We note that Figure 1 in your submission contain [map/satellite] images which may be copyrighted. All PLOS content is published under the Creative Commons Attribution License (CC BY 4.0), which means that the manuscript, images, and Supporting Information files will be freely available online, and any third party is permitted to access, download, copy, distribute, and use these materials in any way, even commercially, with proper attribution. For these reasons, we cannot publish previously copyrighted maps or satellite images created using proprietary data, such as Google software (Google Maps, Street View, and Earth). For more information, see our copyright guidelines: http://journals.plos.org/plosone/s/licenses-and-copyright.  We require you to either (1) present written permission from the copyright holder to publish these figures specifically under the CC BY 4.0 license, or (2) remove the figures from your submission:  a. You may seek permission from the original copyright holder of Figure 1 to publish the content specifically under the CC BY 4.0 license.  We recommend that you contact the original copyright holder with the Content Permission Form (http://journals.plos.org/plosone/s/file?id=7c09/content-permission-form.pdf) and the following text: “I request permission for the open-access journal PLOS ONE to publish XXX under the Creative Commons Attribution License (CCAL) CC BY 4.0 (http://creativecommons.org/licenses/by/4.0/). Please be aware that this license allows unrestricted use and distribution, even commercially, by third parties. Please reply and provide explicit written permission to publish XXX under a CC BY license and complete the attached form.”  Please upload the completed Content Permission Form or other proof of granted permissions as an "Other" file with your submission.  In the figure caption of the copyrighted figure, please include the following text: “Reprinted from [ref] under a CC BY license, with permission from [name of publisher], original copyright [original copyright year].”  b. If you are unable to obtain permission from the original copyright holder to publish these figures under the CC BY 4.0 license or if the copyright holder’s requirements are incompatible with the CC BY 4.0 license, please either i) remove the figure or ii) supply a replacement figure that complies with the CC BY 4.0 license. Please check copyright information on all replacement figures and update the figure caption with source information. If applicable, please specify in the figure caption text when a figure is similar but not identical to the original image and is therefore for illustrative purposes only. The following resources for replacing copyrighted map figures may be helpful:  USGS National Map Viewer (public domain): http://viewer.nationalmap.gov/viewer/ The Gateway to Astronaut Photography of Earth (public domain): http://eol.jsc.nasa.gov/sseop/clickmap/ Maps at the CIA (public domain): https://www.cia.gov/library/publications/the-world-factbook/index.html and https://www.cia.gov/library/publications/cia-maps-publications/index.html NASA Earth Observatory (public domain): http://earthobservatory.nasa.gov/ Landsat: http://landsat.visibleearth.nasa.gov/ USGS EROS (Earth Resources Observatory and Science (EROS) Center) (public domain): http://eros.usgs.gov/# Natural Earth (public domain): http://www.naturalearthdata.com/](https://url.au.m.mimecastprotect.com/s/Wyt7C71R2NTQE33x1t2FnSorY73?domain=track.editorialmanager.com) | We confirm we have obtained written permission from Lismore City Council to include Figure 1 in our submission and we have edited the Figure caption as required. Please see attached for the completed Content Permission Form. |
| If the reviewer comments include a recommendation to cite specific previously published works, please review and evaluate these publications to determine whether they are relevant and should be cited. There is no requirement to cite these works unless the editor has indicated otherwise. | N/A |
| Please review your reference list to ensure that it is complete and correct. If you have cited papers that have been retracted, please include the rationale for doing so in the manuscript text, or remove these references and replace them with relevant current references. Any changes to the reference list should be mentioned in the rebuttal letter that accompanies your revised manuscript. If you need to cite a retracted article, indicate the article’s retracted status in the References list and also include a citation and full reference for the retraction notice. | We confirm we have reviewed our reference list for completeness and correctness. No retracted papers have been cited in our manuscript. |
