# Supplementary material for: Housing displacement experiences following major flooding in regional Australia: A qualitative study of online news articles
Source: PLoS One. 2026 Sep 9;21(9):e0357805. doi: 10.1371/journal.pone.0357805 (PMC13557363; doi:10.1371/journal.pone.0357805)
Supplement: Supplementary file 6 [file pone.0357805.s007.docx]

## Response to Reviewers (Round 2)

**Paper for PLOS ONE**

**Paper:** Housing displacement experiences following major flooding in regional Australia: a qualitative study of online news articles

**Authors:** Jessica Chen, Rebecca McNaught, Jodie Bailie, Kanchana Ekanayake, Ross Bailie

Thank you to the reviewers and editors for their second round of feedback. As there were no request for changes from Reviewer 1, the table below addresses feedback from Reviewer 2 only. We note that changes to the manuscript in response to feedback from Reviewer 2 are provided in track changes made within the clean version of our manuscript from our second submission made on 16 May 2026.

We would like to note that in our resubmitted manuscript (16 May 2026) we replaced some of our tables with figures and moved the corresponding tables to our supporting information files. This was in response to the first round of reviewer feedback regarding presentation of data. We note that some of the feedback from Reviewer 2 in this second round refers to tables which are no longer included within the main manuscript. We have made note of specific discrepancies below where relevant. While we have attempted to identify each table through context, we note there may be inconsistencies.

| **Reviewer 2 Feedback** | **Response** |
| --- | --- |
| The article addresses an important social challenge of housing displacement after flooding. Health and equity implications were drawn from a qualitative analysis of news articles and the gaps were equally highlighted. There is coherency in the narrative, but for consistency and clarity, the areas to be strengthened include: | Thank you for your feedback. Please see below for the changes we have made in response. |
| 1. Tables   In Table 1 - check whether ‘World Socialist Wide Web’ is World Socialist Website’.  Table 2 - the footnote can be moved into the caption for readability. Bolden the highest values in each row to highlight trends. It will be more appropriate to use the same time interval (six months).  Table 3 - clarify overlaps and ensure consistency e.g., caravans, Pod villages, and Temporary pods.  Table 4 – vulnerable populations (Indigenous people, disabled, single parents, and elderly) can be grouped together under discussion for emphasis.  Table 5 - Mental health descriptors can be put together into categories to aid interpretation. | Table 1 - Thank you for picking this up, we have amended ‘World Socialist Wide Web’ to ‘World Socialist Website’ and confirmed this outlet is correctly named within Supplementary Information File 1 (S1 File).  Table 2 - we note that, in addressing the first round of feedback, Table 2 was amended to Figure 2 within our main manuscript and now appears as Table A in Supplementary Information File 2 (S2 File) within our second submission on 16 May 2026.  Where relevant, we have moved footnotes to table captions to improve readability.  Regarding the time intervals selected, please see our comment below addressing Point 2 (Figures).  Table 3 - we note that, in addressing the first round of feedback, Table 3 was amended to Table 2 within our revised manuscript submitted on 16 May 2026. After reviewing this table, we have combined accommodation measures that overlap where appropriate, and grouped similar measures together to allow better comparison (see track changes). We have also moved the footnote to the caption below the table to improve readability.  Table 4 & Table 5 - we note that in response to previous feedback that recommended we utilise other methods of presenting data, the following changes were made in our revised submission from 16 May 2026:  - Table 4 was amended to Figure 4 and 5 within our main manuscript and appears as Table B in the supplementary File (S2)  - Table 5 was amended to Figure 6 within our main manuscript and appears as Table C in S2 File.  Given Tables 4 and 5 have been moved to supplementary information, we do not feel these changes would add value to our main manuscript, and therefore we have not updated the corresponding tables within S2 File. Differentiating vulnerable populations (rather than grouping them) is useful for identifying specific social vulnerabilities and population groups that require future research and support services. Note that mental health descriptors were listed as they appeared in newspaper articles in order to ascertain the frequency of words. We feel it is useful for analysing disaster related discourse to maintain the detail of words as they occurred in the newspaper articles rather than aggregating them. |
| 2. Figures   Use 6-month intervals in Figure 2 | Thank you for your feedback regarding using 6 months intervals within Figure 2 and Table A in S2 File (previously Table 2 in our first submission).  As noted within our manuscript, two flooding events occurred in Lismore in 2022 approximately one month apart. Period 1 represents this critcal one month period between the two flooding events, with Periods 2, 3, 4 and 5 representing subsequent 6-month disaster recovery intervals following the second flood event.  We feel it is important to maintain the time intervals as explained above within relevant figures and tables, and as referenced within the results of our main manuscript, as Period 1 represents a distinct time period that is important to distinguish from subsequent periods of news reporting.  To avoid confusion for readers, we have included a footnote in the caption of Figure 2 and Table A regarding the above, justifying this differentiated time period and avoiding confusion for readers (see track changes). |
| 3. Overall Recommendation:   Shorten the abstract sentences and concentrate on the novelty of using news articles as a qualitative source.  In the discussion, expand on the policy implications, that is, how government can better integrate informal housing arrangements into disaster planning.  Point out the limitations of the study, such as potential bias of the media reporting.  Let the conclusion be directed more to policy makers and researchers as a wake-up call to action.  All in all, the paper is solid and publishable. The authors should make the adjustments with respect to the tables and figure. Expanded discussion on vulnerable groups and policy implications will strengthen the relevance of the article. | Thank you for your feedback. We have addressed these comments as suggested within our manuscript (see track changes).  In the Abstract (noting that we are confined by a 300 word limit)  Discussion (noting policy and legislative gaps in Australia)  Limitations (noting potential bias of media reporting)  Conclusion (policy and legislation recommendations).  Our discussion highlights the discrepancies between media reporting and issues highlighted by local organisations post flood – indicating the limitations of news analysis and lack of focus on vulnerable groups by media. |
